# Non-IgE-Mediated Gastrointestinal Food Protein-Induced Allergic Disorders. Clinical Perspectives and Analytical Approaches

**DOI:** 10.3390/foods10112662

**Published:** 2021-11-02

**Authors:** Elisa Zubeldia-Varela, Tomás Clive Barker-Tejeda, Frank Blanco-Pérez, Sonsoles Infante, José M. Zubeldia, Marina Pérez-Gordo

**Affiliations:** 1 Department of Basic Medical Sciences, Facultad de Medicina, Institute of Applied Molecular Medicine (IMMA), Universidad San Pablo-CEU, CEU Universities, ARADyAL, 28660 Madrid, Spain; elisa.zubeldiavarela@ceu.es (E.Z.-V.); tomas.barkertejeda@ceu.es (T.C.B.-T.); 2 Centre for Metabolomics and Bioanalysis (CEMBIO), Department of Chemistry and Biochemistry, Facultad de Farmacia, Universidad San Pablo-CEU, CEU Universities, Urbanización Montepríncipe, Boadilla del Monte, 28660 Madrid, Spain; 3 VPr1 Research Group “Molecular Allergology”, Paul-Ehrlich-Institut, Federal Institute for Vaccines and Biomedicines, 63225 Langen, Germany; frank.blanco@pei.de; 4 Allergy Paediatric Unit, Allergy Service, Hospital General Universitario Gregorio Marañón, 28007 Madrid, Spain; sonsolesinfante@yahoo.es (S.I.); josemanuel.zubeldia@salud.madrid.org (J.M.Z.); 5 Gregorio Marañón Health Research Institute (IiSGM), 28007 Madrid, Spain; 6 Rare Diseases Networking Biomedical Research Centre (CIBERER, U-761), Carlos III Institute of Health, 28029 Madrid, Spain

**Keywords:** food allergy, non-IgE-mediated gastrointestinal food allergy, cow’s milk, food protein-induced enterocolitis syndrome, food protein-induced allergic proctocolitis, food protein-induced enteropathy, fecal calprotectin, omics

## Abstract

Non-IgE-mediated gastrointestinal food allergy (non-IgE-GI-FA) is the name given to a series of pathologies whose main entities are food protein-induced allergic proctocolitis (FPIAP), food protein-induced enteropathy (FPE), and food protein-induced enterocolitis syndrome (FPIES). These are more uncommon than IgE-mediated food allergies, their mechanisms remain largely unknown, and their diagnosis is mainly done by clinical history, due to the lack of specific biomarkers. In this review, we present the latest advances found in the literature about clinical aspects, the current diagnosis, and treatment options of non-IgE-GI-FAs. We discuss the use of animal models, the analysis of gut microbiota, omics techniques, and fecal proteins with a focus on understanding the pathophysiological mechanisms of these pathologies and obtaining possible diagnostic and/or prognostic biomarkers. Finally, we discuss the unmet needs that researchers should tackle to advance in the knowledge of these barely explored pathologies.

## 1. Digestive Manifestations of Food Allergy

Food allergy (FA) is a specific immunological response that occurs reproducibly on exposure to a given food. This immune reaction can be due to an IgE-mediated mechanism or a non-IgE-mediated mechanism. IgE-mediated FA is much more common than non-IgE-mediated FA, the symptoms are easily recognized, and the underlying mechanisms are better established [1].

Although the relationship between food allergy and gastrointestinal dysfunction has been known for several years, the exact mechanisms involved have not yet been clearly defined. The diagnosis of non-IgE-mediated gastrointestinal food allergy (non-IgE-GI-FA) is clinical and—due to a lack of a specific biomarker—not always easy. The diagnosis is based on compatible symptoms, the exclusion of other diseases, resolution of the symptoms once the offending food is removed from the diet, and the reappearance of the symptoms when it is reintroduced.

Non-IgE-mediated gastrointestinal food allergy typically comprises three entities: (i) food protein-induced allergic proctocolitis (FPIAP); (ii) food protein-induced enteropathy (FPE), and (iii) food protein-induced enterocolitis syndrome (FPIES).

### 1.1. Food Protein-Induced Allergic Proctocolitis (FPIAP)

Food protein-induced allergic proctocolitis (FPIAP) is a non-IgE-mediated food allergy characterized by bloody stools in a healthy infant. The symptoms begin within the first months of life; usually in the first two months. It affects both exclusively breastfed infants and formula-fed infants; although it appears to be slightly more common in exclusively breastfed infants (60%) [2].

The most common offending triggers are cow’s milk (CM) proteins (76%), followed by hen’s egg (16%), soy (6%), and corn (2%). In 8% of the cases, the etiology could be multiple [3]. Any food can induce FPIAP and, once the food is removed from the diet, symptoms disappear within a fortnight. Therefore, if the symptoms persist, it is necessary to continue withdrawing food or looking for another diagnosis [4].

The pathophysiological mechanism of FPIAP is not well established. It seems that it involves CD8^+^ lymphocytes, Th-2 type lymphocytes, and eosinophil infiltrate in all layers of the colonic mucosa [5]. Additionally, some studies suggest the involvement of T-cells in the pathogenesis of this entity, associated with secretion of tumor necrosis factor-alpha (TNF-α) by activated lymphocytes [6].

The typical clinical manifestations are mucus bloody stools in a healthy appearing infant. The bleeding is usually visible although it can appear as occult blood in the stools. Some patients have iron-deficiency anaemia and less frequent hypoalbuminemia and peripheral eosinophilia [7]. Symptoms such as vomiting, frank diarrhoea, or unwell appearance are not present, unlike FPE and FPIES.

FPIAP is a very common cause of rectal bleeding in infants although the real prevalence is unknown. The population prevalence in infants is approximately 1% to 2%. The prevalence among infants with rectal bleeding ranges from 18% to 64% [8]. Elizur et al. found an accumulative incidence in 13,019 newborns over a two-year period of 0.16% [9]. Other studies have reported that FPIAP represents between 18% and 64% of children with rectal bleeding when the diagnosis is based on withdrawal of the offending food or by biopsy findings from the flexible sigmoidoscopy [10,11].

The diagnosis is clinical and based on the following criteria: (a) small amount of rectal bleeding in an otherwise healthy infant; (b) disappearance of symptoms after all causal food is removed from diet; and (c) exclusion of other causes of rectal bleeding [12]. Although the histopathological findings are not pathognomonic of the disease, Odze et al. suggest that the finding of ≥60 eosinophils/10 high-power fields is enough to establish the diagnosis in 97.4% of the cases [13].

The differential diagnosis includes anal fissure, which is the most common cause of rectal bleeding in children under 12 months; swallowed maternal blood, or other diseases, such as enteric infection, malrotation with volvulus or Hirschsprung disease—but these usually affect the general condition [8].

FPIAP can be an over-diagnosed entity, which is why some authors advocate the performance of an early oral food challenge (OFC) to avoid unnecessary elimination diets [14].

The prognosis is excellent and nearly all infants outgrow the disease by 1 to 3 years of age [15].

### 1.2. Food Protein-Induced Enteropathy (FPE)

Food protein-induced enteropathy (FPE) is also a non-IgE-GI-FA. It is probably the most forgotten disorder of the three, and in recent years there are hardly reports of FPE in the literature. The symptoms begin in the first year of life and manifest as chronic diarrhea and intermittent vomiting. If the symptoms persist, they will lead to poor weight gain, failure to thrive, and villous atrophy. Some children develop an iron-deficiency anemia associated with the presence of occult blood in the stools [16]. The pathophysiologic mechanisms are unknown, as is the real prevalence.

The most common offending triggers are CM proteins and soy. The diagnosis is based on a compatible clinical history, the anatomopathological findings, and the disappearance of the symptoms once the food is removed from the diet. Intestinal lesions are patchy with villus atrophy, crypt hyperplasia, increased number of intraepithelial lymphocytes, and eosinophilic infiltration [12].

The main differential diagnosis is with celiac disease. Clinically and pathologically, they are indistinguishable, but the typical celiac disease antibodies are absent in FPE [17].

### 1.3. Food Protein-Induced Enterocolitis Syndrome (FPIES)

FPIES has been considered a rare disease, whose cumulative incidence rates are between 0.15–0.7% [18]. However, it might be under-diagnosed since the epidemiological data are scarce and variable.

FPIES is a non-IgE-GI-FA and is considered a heterogenous disorder with a wide spectrum of clinical phenotypes. These are based on: (i) time and duration of the symptoms, (ii) age of presentation, and (iii) specific IgE-positive of the causal food. Thus, we can classify FPIES into acute, chronic, later onset, and atypical [19].

Acute FPIES usually presents in infancy, with repetitive protracted vomiting that begins 1 to 4 h after food ingestion. Vomiting is often accompanied by lethargy and pallor and can be followed by diarrhea. Some patients have an isolated fever peak and severe cases can progress to hypothermia, methemoglobinemia, acidemia, and hypotension, mimicking sepsis [20]. Children are well between episodes and have normal growth, and these typical symptoms commonly disappear within 24 h.

Chronic FPIES is less well characterized. In this presentation, the symptoms occur over several days or weeks and do not have a clear relationship with the offending food. Children have intermittent vomiting and/or diarrhea, failure to thrive, or poor weight gain. The severe form can develop dehydration and hypovolemic shock. In chronic FPIES, once the offending food is removed from the diet, the symptoms subside within two weeks, but re-exposure to it triggers the typical symptoms of acute FPIES. When the disease onsets in adolescence or adulthood, the symptoms appear after years of tolerance to the food and the predominant symptoms are, in most cases, colic abdominal pain and diarrhea [19,20,21,22].

Although FPIES is considered a non-IgE-mediated FA, in some patients, it is possible to detect specific IgE against the causal food, either at the beginning or during the course of the disease. This form of presentation is known as atypical FPIES and it seems to have a protracted course, with a further risk of developing IgE-mediated symptoms. In adults, it has been also described that severe FPIES phenotypes developed IgE and this is linked to the worst prognosis of the disease [19,21,23,24,25,26].

Any food can trigger FPIES; however, there are geographical differences, and some foods are more prevalent than others. In the USA, the most causative foods are CM proteins and soy. Children with either CM- or soy-induced FPIES can also react to both foods and have an increased likelihood of reacting to a solid food, most commonly oats and rice. In Asia, CM, soy-based formulas, and rice are the most common causal foods. Soy is especially frequent in Korea, but it has a better prognosis and earlier resolution than in other countries. Rice has been observed to be the main trigger of FPIES among the Australian pediatric population, and in European countries, CM and fish are the most frequent triggers of FPIES [27,28,29].

The development of tolerance varies by type of trigger and geographic location. CM- and soy-induced FPIES are reported to occur at an earlier age than other foods. The average age of tolerance of grains-FPIES is 35 months and fish-FPIES usually resolves within the first 5 years [27,28].

Nowadays, there is not a specific biomarker for FPIES that allows for confirmation of the diagnosis or to predict when the tolerance to the offending food has been achieved. For this reason, all patients with FPIES should undergo an oral food challenge (OFC) at least once in life. The OFC is a risky procedure and patients can develop moderate or severe symptoms after it, so it should be always performed under medical supervision [20,30].

**Figure 1:**
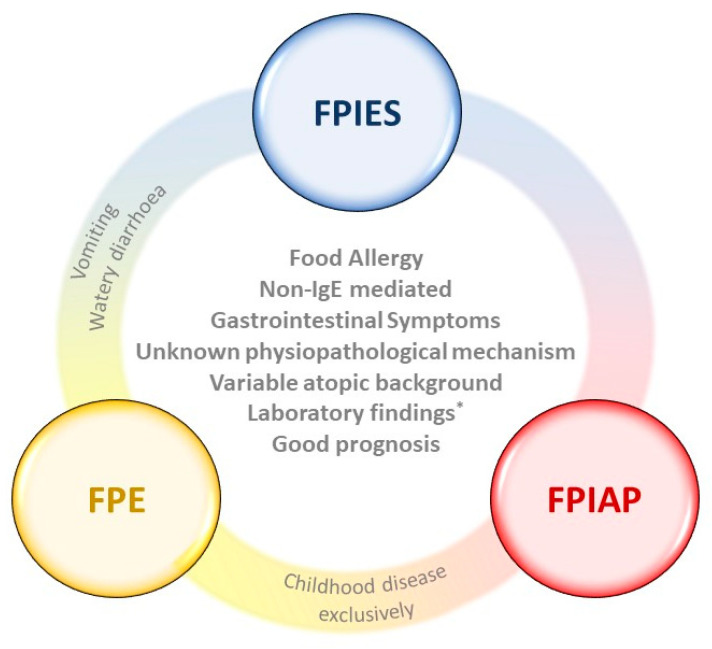
Description of differences and similarities between FPIES, FPE, and FPIAP. FPIES: food protein-induced enterocolitis syndrome; FPE: food protein-induced enteropathy; FPIAP: food protein-induced allergic proctocolitis. * Laboratory findings (e.g., anemia, iron deficiency, eosinophilia, rectal bleeding, etc.) are similar as long as the food is not withdrawn from the diet.

The diagram shown in Figure 1 summarizes similarities and differences of FPIAP, FPE, and FPIES.

## 2. Approaches for the Study of Non-IgE-GI-FA

The most important diagnostic tool in the evaluation of non-IgE-GI-FA is a careful clinical history. However, when the clinical history is not sufficiently thorough, the OFC should be used as the gold standard to confirm the diagnosis of this disease, as has been mentioned above [21,31,32].

There are no other specific diagnostic procedures for these diseases, mainly because their pathophysiological mechanisms are poorly understood. However, there are a variety of laboratory tests to support the diagnosis and, more importantly, to rule out other conditions such as lactose intolerance, sepsis, necrotizing enterocolitis, anaphylaxis, gastrointestinal reflux disease, and celiac disease, among others [20]. In this sense, animal models are also playing an important role in the understanding of the pathophysiological mechanisms involved in these pathologies.

**Figure 2:**
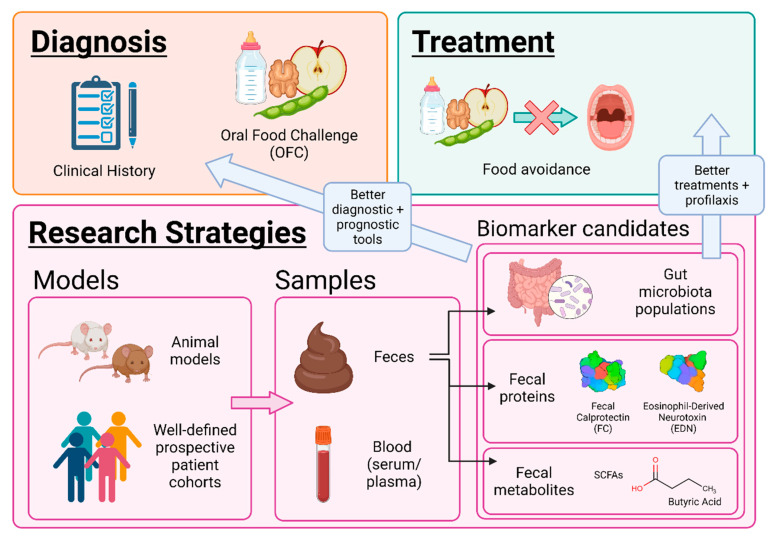
Approaches for the study of non-IgE-mediated gastrointestinal food allergy. SCFAs: short-chain fatty acids.

For the in-depth study of non-IgE-GI-FA, feces are the sample of choice. This is due to the fact that the main system affected is the gastrointestinal tract and to their non-invasive collection [33]. There are fecal biomarkers, such as immune cell derived proteins and/or gut microbiota metabolites, defined as biological entities capable of predicting an individual’s physiological state or pathological condition. The detection of these biomarkers by analytical techniques is on the rise to learn more about the underlying mechanisms of these pathologies [16,34]. In this review, different emerging approaches, such as animal models, omics techniques, and identification of fecal biomarkers are described to deepen the understanding of non-IgE-GI-FA (Figure 2).

### 2.1. Animal Models

Animal models are a useful tool to study allergic diseases in vivo. Several mouse models have been developed to study the gastrointestinal reactions related to food allergy [35,36,37,38,39,40,41,42,43]. Unfortunately, while several IgE-mediated food allergy models have been developed [35,41,42,43,44,45], the generation of non-IgE-mediated murine models has been harder to accomplish. For example, to our knowledge, no animal models have been successfully developed for FPIES, FPIAP, and FPE. However, Burggraf et al. generated a mouse model with intestinal symptoms of a food allergy which resemble these pathologies to study their pathomechanism. This model is known as allergic enteritis (AE), and it mimics in mice some traits observed in patients with non-IgE-GI-FA, such as intestinal inflammation characterized by thicker basal layer, crypt elongation, villous atrophy, and granulocyte infiltration [37]. The initial stimulus leading to the development of allergic enteritis remains to be elucidated, but it is postulated that delayed Th2 cell mediated responses are involved in the development of the inflammation [21,23,24,25,26]. In this model, mice are intraperitoneally sensitized with ovalbumin (OVA) and alum as adjuvant and then challenged with a diet containing high amounts of egg white (EW-diet) [37]. After 4 weeks, tolerance is achieved, but chronic inflammation is still present, despite the lack of allergic signs and the OVA-specific IgE reduction [37]. In a recent study, this AE murine model was used to elucidate the molecular and cellular mechanisms underlying AE [46]. A CC chemokine receptor 8 (CCR8) knock-out mouse was used. CCR8 deficiency translated in a reduced inflammatory profile, characterized by a reduction in the number of infiltrating eosinophils and an enhanced number of neutrophils in the lamina propria of the CCR8KO mice [46]. This revealed that the chemokine CCL1 and its receptor CCR8 are essential for the recruitment of eosinophils in AE sites [46].

In another model, OVA23-3 mice, which express an OVA-specific T-cell receptor, were used to demonstrate that Peyer’s patches (PPs) and mesenteric lymph nodes (MLNs) promote intestinal inflammation in AE [47]. This is through an enhanced infiltration of CD4+ cells in the MLNs, and IL-4 production in the PPs, which decreased in mice when the MLNs and PPs were surgically removed [47].

Closely related to non-IgE-GI-FA is eosinophilic esophagitis (EoE), which is a chronic allergic disease of the esophagus. A mix of immediate, IgE-mediated and delayed, non-IgE-mediated immunological reactions to foods is thought to play a role in EoE [48]. EoE murine models are the best characterized of this group of diseases and have been crucial to characterize the pathology [40,49,50,51].

The use of animal models has been crucial to the understanding not only of the pathology associated with the disease but also to the pre-clinical evaluation of new therapeutics. That makes it of high importance in the development of new and more accurate models that allow a better understanding of non-IgE-GI-FAs.

### 2.2. Gut Microbiota and Omics Techniques

The human microbiota and its genetic composition—known as the human microbiome—began to be studied on a large scale at the beginning of the 21st century within the framework of major projects such as the Human Microbiome Project [52]. Among the microbial ecosystems of the human body, the gut microbiota, present in the gastrointestinal tract, is considered a highly important “superorganism” due to its diversity and complexity [53,54].

Approximately 70% of the primary colonization of the gut microbiota of newborns is vertically transmitted, occurring directly from mother to offspring. However, the composition of the gut microbiota is dynamic and changes throughout life due to factors such as delivery mode, age, diet, antibiotic consumption, ethnicity, lifestyle, environmental factors, and diseases and disorders, among others [33,55,56,57].

**Figure 3:**
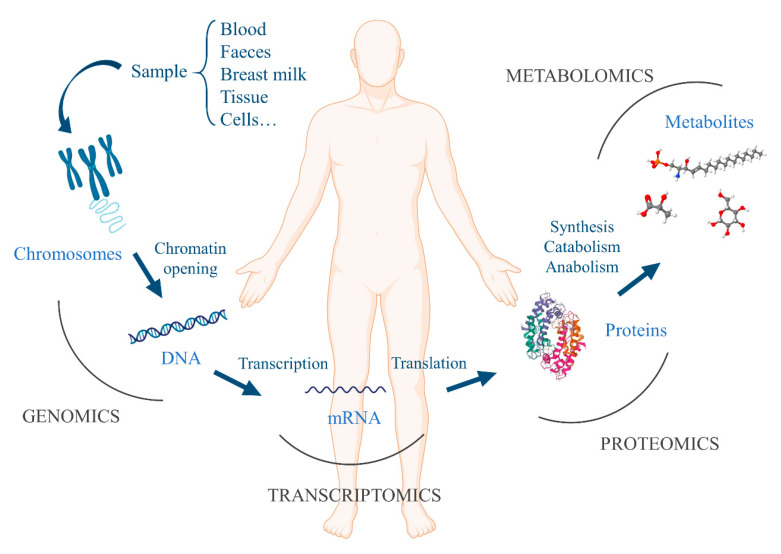
Main omics techniques: genomics, transcriptomics, proteomics, and metabolomics.

The development of the gut microbiota during the first years of life correlates with the development and maturation of the immune system. Moreover, the gut microbiota and the production of gut microbiota-derived short-chain fatty acids (SCFAs: acetic, propionic, and butyric acids) have been shown to be crucial for the proper expression and function of regulatory T cells (Treg), which are important in the induction and maintenance of oral tolerance to food antigens in humans [58,59,60,61,62,63,64,65]. Trompette et al. described in 2014 how high-fiber diet intake in mice offered protection against allergic inflammation in the lungs due to an increased level of circulating SCFAs compared to normal diet-fed mice [66]. Bunyavanich et al. studied the association between CM allergy resolution and early-life gut microbiota, showing an association between gut microbiome at early stages of life (from 3 to six months of age) and milk allergy resolution by age 8 years. This fact was due to the presence of bacterial taxa Clostridia and Firmicutes, suggesting them as potential probiotics candidates for milk allergy treatment [61]. For the analysis and understanding of the gut microbiota, omics are the techniques of election. In the last decades, advances in bioinformatics, as well as the development of high-throughput technologies, have contributed to the development of omics sciences for the study of multifactorial diseases, including allergy [67]. This group of sciences encompasses different disciplines such as genomics (the genome and its function), transcriptomics (mRNA and gene expression), proteomics (structure, function, location, and interaction of proteins), and metabolomics (metabolites and metabolism) (Figure 3) [68].

All the omics are based on the analysis of a large volume of data, and they have allowed a paradigmatic change in the development of research strategies [67,69,70,71]. In this context, a multi-omics approach offers the best opportunity to understand the mechanisms that underlie a disease, giving an overall biological meaning. However, the gut microbiota of non-IgE-GI-FA remains poorly characterized, and few authors have used omics techniques to explore it. So far, only studies using genomics (16S rRNA-gene sequencing) and metabolomics (SCFAs detection) have been published, as discussed below.

Gut microbiota composition and fecal butyrate levels in children affected by non-IgE-mediated CMA were evaluated by Berni Canani et al. [63]. The authors described elevated relative abundances of two Bacteroidetes genera, *Bacteroides* and *Alistipes*, and a single Firmicutes, *Sarcina*, at diagnosis, in children with non-IgE-mediated CMA when compared to healthy controls. However, at the phylum level, only Bacteroidetes was significantly enriched in children with non-IgE-mediated CMA. In addition, the authors observed that these children had a significantly lower fecal concentration of butyrate compared to healthy controls.

Furthermore, Díaz et al. analyzed the composition of gut microbial communities and fecal associated parameters in 17 infants with non-IgE-mediated CMA (under a milk elimination diet) compared to 10 healthy infants (with an unrestricted conventional diet) to establish potential links between the type of formula substitutes, gut microbiota, and desensitization [72]. In this study, all participants received an exclusion diet for six months consisting of extensively hydrolyzed formula (EHF) (12 patients, 70.6%), soy protein-based formulas (2 patients, 11.8%), and hydrolyzed rice formulas (3 patients, 17.6%). Díaz et al. observed that non-allergic infants with unrestricted diet showed a significantly higher proportion of *Bacteroides* compared to infants with non-IgE-mediated CMA. In contrast, some members of the *Clostridiales* order, such as Lachnospiraceae and Coriobacteriaceae families, were significantly higher in allergic infants. In addition, children fed with hydrolyzed rice formulas were the only ones which did not develop tolerance to CMA and the microbiota colonization pattern was characterized by a low abundance of sequences of Actinobacteria, in particular the genus *Bifidobacteria*. Regarding metabolomics, two branched-chain fatty acids (BCFAs: isobutyric and isovaleric) presented significantly higher levels in allergic infants, but SCFAs were not statistically significant.

Looking further into feeding, Candy et al. investigated the effect of a synbiotic-containing (prebiotic blend of fructo-oligosaccharides and the probiotic strain *Bifidobacterium breve* M-16V) amino acid-based formula (AAF) in patients with non-IgE-mediated CMA [73]. Infants fed with AAF, which is reserved for those severe cases not responding to an EHF, were taken as the control group. Subjects given AAF including synbiotics had higher *Bifidobacteria* and lower *Eubacterium rectale/Clostridium coccoides* percentages compared with those in the control group—thus, resembling healthy breastfed infants. Differences in gut microbiota were maintained in these patients over a 26-week follow-up period [74]. Furthermore, in a subsequent paper, the 16S rRNA-gene sequencing revealed that the gut microbiota was substantially affected across time [75]. Wopereis et al. observed increased relative abundances in the test versus control in species of Bifidobacterium and Veillonella families and decreased relative abundance in species of the Lachnospiraceae family and *Ruminococcus* and *Alistipes* genera [75]. The healthy breastfed infants showed the lowest average diversity. However, they showed ratios close to the test group in *Bifidobacterium* spp. and *Lachnospiraceae* spp. Finally, the authors observed an increase of L-lactate and a decrease of valeric and isobutyric acids in the test versus control.

In this regard, Guadamuro et al. analyzed changes in gut microbiota composition, before and after milk challenges, from infants with non-IgE-mediated CMA consuming hypoallergenic hydrolyzed formulas [76]. One month after the introduction of intact proteins and dairy products in the diet, the relative abundance of several bacterial groups that belong to the Lachnospiraceae and Ruminococcaceae families decreased, and some fecal lactic acid bacteria (genus *Lactobacillus*) increased. Microbial metabolites such as BCFAs and p-cresol were lower. However, skatole, a microbial degradation product from tryptophan, was significantly increased.

The recently published study by Aparicio et al. aimed to evaluate the fecal microbiome and different immunological parameters in the feces of infants with gastrointestinal disorders and their maternal milk [77]. A cohort of 30 mother–infant pairs, in which the infants were diagnosed with colic (*n* = 12), non-IgE mediated-CMA (*n* = 5), proctocolitis (*n* = 5), or included as healthy controls (*n* = 8), were recruited. The concentrations of Eggerthellaceae, Lachnospiraceae, and Peptostreptococcaceae (at family level) and of *Rothia* (at genus level) was higher in fecal CMA samples than in those from other groups. In contrast, the abundance of *Bifidobacterium* genus decreased in the CMA group. *Erysipelatoclostridium* abundance was higher in controls and *Intestinibacter* was higher in the proctocolitis group followed by the CMA one. Regarding the bacterial composition of breast milk, the relative abundance of the family *Eggerthellaceae* was significantly higher in CMA than in the control and colic groups and *Prolixibacteraceae* was higher in the proctocolitis group than in the CMA group.

The trend in publications of studies analyzing the intestinal microbiota through omics techniques in patients with non-IgE-GI-FA is growing. For this reason, this field is likely to evolve very rapidly in the coming years. Table 1 lists all the studies to date and summarizes what has been found in terms of genomics and metabolomics.

### 2.3. Fecal Biomarkers. Protein Analysis

The attempts to evaluate the use of fecal biomarkers for the diagnosis of non-IgE FAs have been reviewed recently [16]. The main candidates are fecal calprotectin (FC), α-1 antitrypsin, β-defensin, tumor necrosis factor-α (TNF-α), fecal IgA, eosinophil-derived neurotoxin (EDN), and eosinophilic cationic protein (ECP). From these, FC has been the most discussed, and it has been the subject of a recent systematic review for its use as a biomarker for CMA, including IgE- and non-IgE-mediated forms [78].

Calprotectin is an immunomodulatory, antimicrobial, and antiproliferative protein that is present in the cytoplasm of neutrophils, in the membranes of macrophages, in activated monocytes, and in mucosal epithelial cells [79]. The measurement of FC has been evaluated as a non-invasive marker of gastrointestinal inflammation, as it is known that its concentrations are correlated with the level of intestinal mucosal inflammation [80]. Based on this fact, it has been used for the monitoring of intestinal conditions such as inflammatory bowel disease (IBD) [16,81] and distinguishing it from irritable bowel syndrome (IBS) [82]. As for its measurement for CMA, a remarkable example is the study by Beşer and collaborators, which analyzed children with CMA (24 IgE-mediated, 8 non-IgE-mediated) and compared them to 39 controls of the same age range, before and after an elimination diet [83]. These authors found that FC levels were highest in children with non-IgE-mediated CMA, from which they suggest its use as a biomarker. However, the evidence is conflicting, since other authors found no significant differences [72,84]. Systematic reviews have concluded that the available evidence is not enough to encourage its use as a biomarker for monitoring CMA, neither IgE- nor non-IgE-mediated [16,78]. Altogether, authors agree that more well-defined studies are needed, and stress the need for establishing the exact cut-off values of FC in different groups taking into account factors such as age, feeding patterns, clinical symptoms, and immune mechanism type [78].

On the other hand, α-1 antitrypsin is an inhibitor of serine proteases present in serum which is synthesized in the liver [85]. It can be measured to assess the excessive GI protein losses in patients with protein-losing enteropathy (PLE) [86,87]. It should be noted that PLE must not be confused with α-1 antitrypsin deficiency, a separate condition, as has sometimes been reported in the literature [87]. In the case of CMA, there is very little data: to the best of our knowledge, only one paper reports its measurement in a study of CMA, alongside a panel of other markers, and did not find significant differences for this protein [88].

A promising group of proteins as potential biomarkers for CMA are the eosinophilic proteins EDN and ECP. They are proteins released by the degranulation of eosinophils [89] which constitute non-invasive biomarkers of the eosinophilic GI inflammation of non-IgE-GI-FAs [16,90,91].

An interesting example is the work of Kalach et al., who studied the most complete panel of possible biomarkers (FC, α-1 antitrypsin, β-defensin, TNF-α, fecal IgA, EDN, and ECP), as well as intestinal permeability and fecal microbiota, in a pilot study of 11 children with CMA (3 IgE-mediated, 8 non-IgE-mediated) compared to 14 controls. From the panel, they found EDN to be the most promising, highlighting as well the obvious need for studies with a higher population to confirm the results [88]. Other studies analyzing different combinations of these proteins also single out EDN as a potential biomarker for the diagnosis of these pathologies [92,93,94].

## 3. Therapeutical Perspectives

In most cases, FPIAP, FPE, and FPIES have a good prognosis, resolving during childhood. This leads to efforts in the knowledge of pathology to be directed to the search for diagnostic and prognostic biomarkers, relegating the treatment to the withdrawal of the causal food and to the treatment of the acute reaction, in the case of acute FPIES.

For symptomatic infants who are exclusively breastfed, the food should be withdrawn from the maternal diet. For exclusively formula-fed infants, an EHF or AAF should be started. In infants who have already been introduced to other foods and we suspect that one is the cause of the symptoms, the food should be removed from their diet, and tolerance to other foods from the same group should be checked to avoid unnecessarily restricted diets. In the specific case of proctocolitis, if the symptoms are mild, an expectant attitude can be maintained for a few days, since in some cases, a spontaneous resolution occurs without it being necessary to remove the food from the diet [31,95,96].

In the case of an acute reaction in FPIES, the treatment will be staggered according to the severity of the symptoms. Thus, a mild reaction—one or two vomits without lethargy or other symptoms—can be controlled with oral hydration and, optionally, ondansetron. When the reaction is moderate—if the number of vomits is ≥three and is also accompanied by mild lethargy—intravenous fluid therapy should be administered early along with ondansetron. Finally, if the reaction is severe—severe lethargy appears, with hypotonia or cyanosis—in addition to intravenous fluid therapy and ondansetron, a corticosteroid bolus should be administered [20,95,97,98].

## 4. Unmet Needs

There is an ongoing increase in the prevalence of food allergy worldwide, but the cause of this increment remains uncertain [99]. Factors such as pollution, lack of contact with microorganisms, and fewer unprocessed natural products in diet, seem to be crucial in the development of allergies in recent decades [100]. The study and comprehension of the different allergic endotypes is essential to developing precise therapeutic approaches and personalized treatments. In the case of non-IgE-GI-FA, the etiology and the underlying pathological mechanisms, including the involvement of the immune system, are poorly understood. Therefore, it is crucial to develop new diagnostic, prevention, and intervention strategies to manage non-IgE-GI-FA.

The development of non-invasive methods to study immune cells activated by food exposure in the gastrointestinal tract is needed to understand how symptoms are triggered [101]. In this sense, animal models and the identification of fecal biomarkers will be optimal. However, non-IgE-mediated murine models are difficult to generate due to the particularity of these pathologies, and results are not always easy to extrapolate to human. In the case of fecal biomarkers, most authors concur about the fact that there is a lack of exact cut-off values, and that study groups should be better defined, taking into account variables such as age, feeding patterns, clinical symptoms, and type of immune mechanism implicated [16,78].

In this context, the identification of a metabolic fingerprint of non-IgE-GI-FA by the application of the abovementioned omics techniques can lead to the development of better diagnostic tools and/or new treatment options. Indeed, it is expected that with a multiomic approach, a combination of different biomarkers will provide the opportunity to monitor allergic conditions and predict whether a patient will respond to a specific therapeutic approach or not. The study of predictive metabolic biomarkers of the disease could be also reached by these techniques in a near future.

One rising strategy for both prevention and intervention in non-IgE-GI-FA is the use of probiotics, prebiotics, and synbiotics to modulate gut microbiota. In this regard, some authors have described beneficial alterations of the intestinal microbiota by adding to certain diets compounds such as the probiotic *Lactobacillus rhamnosus* GG, and the synbiotic composed of a prebiotic blend of fructo-oligosaccharides and the probiotic strain *Bifidobacterium breve* M-16V to ameliorate non-IgE-mediated CMA symptoms [64,72]. Wopereis H et al., referred the effects of an AAF diet including specific synbiotics on infants with non-IgE-mediated CMA compared to healthy breastfed infants, showing that this specific supplemented diet modulates gut microbiota towards a healthy breastfed profile [75].

To sum up, future efforts in non-IgE-GI-FA research should be aimed at elucidating the underlying pathological mechanisms of the diseases in order to understand the etiology of the symptoms and develop diagnostic and prognostic tools as well as innovative treatment targets.

## Figures and Tables

**Table 1:** Summary of described studies using genomics and metabolomics to study non-IgE-GI-FA.

Disease	Description	Diet of Allergic Infants	Microbial Communities (Genomics)	Metabolites (Metabolomics)	Reference
Non-IgE-mediated CMA	Allergic vs. healthy children	No dietary restriction (collection at diagnosis)	↑ Phylum Bacteroidetes ↑ Genera *Bacteroides*, *Alistipes*, and *Sarcina*	↓ Fecal butyrate	Berni Canani et al. [63]
Non-IgE-mediated CMA	Allergic vs. healthy infants (unrestricted conventional diet)	Milk exclusion diet for six months: ‐ 12 EHF ‐ 2 SPBF ‐ 3 HRF	**EHF, SPBF & HRF:** ↑ Families *Lachnospiraceae* and *Coriobacteriaceae* ↓ Genus *Bacteroides* **HRF:** ↓ Genus *Bifidobacteria*	↑ Isobutyric and isovaleric acids = Acetic, propionic, and butyric acids	Díaz et al. [72]
Non-IgE-mediated CMA	Allergic with test formula vs. allergic with control formula vs. HBI at week 8	Amino acid-based formula for 8 weeks: ‐ 35 test formula (synbiotic-containing) ‐ 36 control formula	*Bifidobacteria*: HBI > test formula > control formula *Eubacterium rectale/Clostridium coccoides*: HBI < test formula < control formula	-	Candy et al. [73]
Non-IgE-mediated CMA	Allergic with test formula vs. allergic with control formula at week 26	Formula for 26 weeks: ‐ 26 test formula (synbiotic-containing AAF) ‐ 28 control formula (AAF) ‐ 2 control formula (CMF)	*Bifidobacteria*: test formula > control formula *Eubacterium rectale/Clostridium coccoides*: test formula < control formula	-	Fox et al. [74]
Non-IgE-mediated CMA	Allergic with test formula vs. allergic with control formula vs. HBI	Amino acid-based formula: ‐ 35 test formula (synbiotic-containing) ‐ 36 control formula	**Test vs. control:** ↑ Species of *Bifidobacterium* and *Veillonella* families ↓ Species of *Lachnospiraceae* family and *Ruminococcus* and *Alistipes* genera **HBI vs. test formula:** ≈ *Bifidobacterium* spp. and *Lachnospiraceae* spp.	**Test vs. control:** ↑ L-lactate ↓ Valeric and isobutyric acids = Acetate, propionate, butyrate, iso-valerate, and D-lactate	Wopereis et al. [75]
Non-IgE-mediated CMA	Allergic children before and after milk challenges	Hypoallergenic hydrolyzed formulas	↑ Lactic acid bacteria (genus *Lactobacillus*) ↓ Bacterial groups of *Lachnospiraceae* and *Ruminococcaceae* families	↑ Skatole ↓ *p*-cresol and BCFAs = Acetic, propionic, and butyric acids	Guadamuro et al. [76]
Gastrointestinal disorders	Colic vs. non-IgE-mediated CMA vs. proctocolitis vs. healthy controls	Exclusive breastfeeding	**Fecal microbiota** in non-IgE-mediated CMA: ↑ Families *Eggerthellaceae*, *Lachnospiraceae*, and *Peptostreptococcaceae* ↑ Genera *Intestinibacter* (more increased in proctocolitis) and *Rothia*↓ Genera *Erysipelatoclostridium* (higher in controls) and *Bifidobacterium* **Breast milk:** ↑ Family *Eggerthellaceae* in non-IgE-mediated CMA ↑ Family *Prolixibacteraceae* in proctocolitis	-	Aparicio et al. [77]

Footnote. CMA: cow’s milk allergy; EHF: extensively hydrolyzed formula; SPBF: soy protein-based formula; HRF: hydrolyzed rice formula; HBI: healthy breastfed infants; AAF: amino acid-based formula; CMF: cow’s milk formula; BCFAs: branched-chain fatty acids. Arrow ↑ stands for increased levels of microbial communities and/or metabolites. Arrow ↓ stands for decreased levels of microbial communities and/or metabolites.

## Data Availability

Not applicable.

## Abbreviations

AAF	amino acid-based formula
AE	allergic enteritis
BCFAs	branched chain fatty acids
CCR8	CC chemokine receptor 8
CM	cow’s milk
CMA	cow’s milk allergy
CMF	cow’s milk formula
ECP	eosinophilic cationic protein
EDN	eosinophil-derived neurotoxin
EHF	extensively hydrolyzed formula
EoE	eosinophilic esophagitis
FA	food allergy
FC	fecal calprotectin
FPE	food protein-induced enteropathy
FPIAP	food protein-induced allergic proctocolitis
FPIES	food protein-induced enterocolitis syndrome
GI-FA	gastrointestinal food allergy
HBI	healthy breastfed infants
HRF	hydrolyzed rice formula
MLNs	mesenteric lymph nodes
non-IgE-GI-FA	non-IgE-mediated gastrointestinal food allergy
OFC	oral food challenge
PPs	Peyer’s patches
SCFAs	short-chain fatty acids
SPBF	soy protein-based formula
TNF-α	tumor necrosis factor-α
